# Intensive trauma-focused treatment for PTSD and Complex PTSD in a public clinic: a feasibility study

**DOI:** 10.3389/fpsyg.2026.1869412

**Published:** 2026-09-09

**Authors:** Julie Horgen Evensen, Jeanette Engeset, Marianne Jakobsen, Anders Malkomsen, Veronica Vaage-Kowalzik

**Affiliations:** 1 Nydalen District Psychiatric Center, Division of Mental Health and Addiction, Oslo University Hospital, Oslo, Norway; 2 Southern Oslo District Psychiatric Center, Division of Mental Health and Addiction, Oslo University Hospital, Oslo, Norway; 3 Private Practitioner, Asker, Norway

**Keywords:** EMDR, intensive trauma-focused treatment, PE, PTSD, therapist rotation, Trauma-focused treatment

## Abstract

**Background:**

Intensive trauma-focused treatment (ITFT) has demonstrated robust results in treating post-traumatic stress disorder (PTSD) in specialized trauma centers. Whether this treatment is feasible in a non-specialized treatment facility has not been previously investigated.

**Objective:**

This pilot study aimed to examine if an ITFT program is feasible in a non-trauma-specialized public treatment facility, where many therapists had limited experience with trauma-focused treatment.

**Methods:**

Eighteen female patients diagnosed with PTSD or Complex PTSD (CPTSD), with multiple traumas and comorbidity, participated in an intensive 2-week (4 + 4 days) inpatient trauma treatment program that included Prolonged Exposure, Eye Movement Desensitization and Reprocessing, group psychoeducation, and physical activity, and that also used therapist rotation. The patients were assessed using a battery of validated self-report questionnaires and interviews before and after the intervention, and at 2 and 6 months post-treatment. Therapists' and ward staff's readiness to provide TFT was assessed with a questionnaire after participating in the program.

**Results:**

All participants completed treatment. Individual session attendance was very high, and no significant adverse effects were reported. The reduction in PTSD symptoms was considerable and sustained, with 83% of the sample showing clinically reliable change at the 6-month follow-up. Half of patients with a CPTSD diagnosis and all patients with a PTSD diagnosis lost their diagnosis. Trauma-related negative cognitions also showed a statistically significant reduction from baseline to the 6-month follow-up. Treatment satisfaction was high, including measures of the individual treatment components, and therapist rotation. Both therapists and ward staff reported increased readiness to start TFT after participating in the project.

**Conclusions:**

This feasibility study indicates that intensive trauma-focused treatment for PTSD and CPTSD is acceptable and associated with symptom improvement in a public treatment facility.

**Trial registration:**

ClinicalTrials.gov identifier: NCT05342480. Date of registration: 2022-04-22.

## 1 Background

Trauma-focused treatments (TFT) have shown significant and sustained effects in treating post-traumatic stress disorder (PTSD) (Weber et al., 2021). However, dropout rates have been considerable (Lewis et al., 2020), and patients have reported high levels of distress in between sessions when therapy is delivered weekly (Eastwood et al., 2021). Intensive trauma-focused treatment (ITFT), commonly operationalized as delivering treatment more than twice weekly, has been developed to counter these obstacles. A systematic review of ITFTs indicates that these intensive treatment models have a higher rate of treatment completion (Sciarrino et al., 2020), and suggests that intensive treatments for PTSD may provide an effective alternative to standard approaches by reducing dropout. A further systematic review reported high levels of patient satisfaction with ITFT and sustained treatment effects up to 12 months post-treatment (Ragsdale et al., 2020).

To further optimize treatment effects, an ITFT combining two psychotherapeutic models targeting PTSD symptoms–Prolonged Exposure (PE) and Eye Movement Desensitization and Reprocessing (EMDR) – has been developed at the Psychotrauma Expertise Center (PSYTREC), Netherlands (Van Woudenberg et al., 2018). The intensive 2-week program also includes physical activity (PA) and psychoeducation groups (PEG) and uses therapist rotation (TR) to deliver therapy (Van Minnen et al., 2018). This clinical research milieu has documented robust treatment results for patients with PTSD, with sustained effects measured up to 6-month follow-up (Van Woudenberg et al., 2018). Substantial improvement has also been shown for patients with PTSD resulting from childhood sexual abuse (Wagenmans et al., 2018) and for patients with comorbid disorders (Kolthof et al., 2022; Paridaen et al., 2023) complex PTSD (CPTSD) (Voorendonk et al., 2020), and the dissociative subtype of PTSD (Zoet et al., 2018). To our knowledge, versions of the Dutch intensive treatment program, using both EMDR and PE, as well as TR, have only been reported in two previous quantitative studies outside the very specialized trauma center in the Netherlands. First, it was successfully adapted to an outpatient facility specialized in trauma-focused treatment in Norway (Auren et al., 2022), with a sample of twenty-nine patients. The authors reported no drop-out and large effect sizes (*d* = 1.38–1.52) on the final follow up 3 months post-treatment. Second, a recent Swedish feasibility study based on a similar intensive treatment program reported significant reductions in PTSD symptoms, and large effect sizes (*d* = 1.00–1.92) (Gahnfelt et al., 2023). Patients stayed in a hospital hotel facility during treatment, which took place in a specialized trauma-treatment outpatient clinic. The sample consisted of 12 participants, of whom one dropped out during the treatment period. Patients were followed up to 3 months post-treatment.

Providing TFT is known to be demanding for therapists, and burnout is well documented in the trauma therapist population (Craig and Sprang, 2010; Sodeke-Gregson et al., 2013). Studies on therapist factors that hinder implementation of TFT have shown that therapists fear that patients may not tolerate the treatment and deteriorate, as one such factor (Finch et al., 2020; Vaage-Kowalzik et al., 2025a). In the TR model several therapists rotate between patients across treatment sessions within the same ITFT program, so that multiple therapists see the same patients, and share the responsibility of their treatment, and patients get to meet a number of different therapists. It was designed by Van Minnen et al. to reduce hindrances to TFT implementation, including therapist avoidance and drift. In a quantitative survey, they found that therapists reported being less likely to drift from the treatment protocol and less fearful of pressing ahead in TFT sessions (Van Minnen et al., 2018). In a recent study on alliance formation in a four-day ITFT program using therapist rotation, De Jongh et al. found that therapeutic alliance increased significantly from early day two to mid-treatment, and that higher prior PTSD symptom scores predicted poorer alliance, while stronger prior alliance predicted greater symptom reduction (Hoogeveen et al., 2026). In an earlier qualitative study within the current feasibility study, we reported that therapists described TR as a valuable learning arena and a way of sharing the burden of responsibility (Vaage-Kowalzik et al., 2025a). Therapists described how experiencing firsthand that PTSD patients with complex trauma histories and comorbidities were able to tolerate intensive treatment made them more secure in their role as trauma therapists.

In sum, there is robust evidence for the treatment effect of ITFT programs. However, the Dutch model combining PE, EMDR, and TR has been examined only rarely outside the very specialized trauma-treatment center in the Netherlands, and follow-up periods in studies outside the Netherlands have been no longer than 3 months. Data on how participating in ITFT program affects therapists are sparse but suggest that ITFT programs make therapists more ready to start TFT. In the current pilot study, we aimed to examine whether an ITFT program combining PE, EMDR, PA, PEG, and TR was feasible in a Norwegian public, non-trauma-specialized treatment facility. We further aimed to examine whether patients with severe PTSD (defined as PTSD with comorbidities and/or CPTSD) would experience a reduction in PTSD symptoms and whether these improvements were sustained up to 6 months post-treatment. Our main hypothesis was that an ITFT program could be delivered with low dropout rates, high levels of patient satisfaction, and significant long-term improvement in trauma symptom scores. We further hypothesized that participating therapists would experience increased readiness to start TFT.

## 2 Methods

### 2.1 Design and ethics

This study was conducted at a public psychiatric clinic in Oslo, Norway, which offers both inpatient and outpatient treatment. The facility treats a wide range of mental illnesses. It is part of the specialist healthcare system and requires a referral from a doctor or psychologist. The Central Norway Regional Ethics Health Committee (REC Southeast 0704/2022) has approved the study. Clinical Trial gov. identifier NCT05342480.

### 2.2 Participants and procedure

Patients were recruited from the outpatient clinic's patient pool. Eighteen patients, in three groups of six, participated in the study. To be included in the study, patients had to: meet diagnostic criteria for PTSD, report more than one traumatic life event, have experienced symptoms for more than 6 months, and have had at least one prior psychotherapeutic treatment attempt (>3 months duration). Patients had to be between the ages of 18 and 65 and be able to speak a Scandinavian language proficiently. Exclusion criteria included psychotic or bipolar disorder, current substance abuse, being in an abusive or life-threatening situation, or a serious suicide attempt within the last 3 months. Before referral, patients underwent diagnostic assessment with the Mini-International Neuropsychiatric Interview (MINI) (Sheehan et al., 1998) and, when indicated, the Structured Clinical Interview for DSM-IV-R (SCID-II) (First, 1997). After referral, patients were invited to an assessment interview, where they received additional information about the treatment before giving informed written consent. Of the 26 referred patients, none were excluded based on the inclusion or exclusion criteria. However, seven changed their minds about participating because current life events or situations made participation difficult (taking care of children (2), exams (2), work situation (1), not feeling ready to engage with TFT (1), pain and somatic illness (1), while one was not currently suitable for engagement in group activities.

### 2.3 Therapists, ward staff and treatment

The treatment program spanned 2 weeks, with eight full treatment days (Monday through Thursday). The program included daily PE and EMDR sessions, with PA and PEG between sessions. It is described in detail in an earlier paper (Vaage-Kowalzik et al., 2024) and in Supplementary Data Sheet 1. Patients attended follow-up sessions and evaluations with the treatment program after 2 and 6 months. All patients remained under the primary care of their outpatient therapists before and after treatment. The first session with their outpatient therapist was scheduled 1 week after discharge. Following this session, the outpatient therapists determined the level of follow-up care. However, they were asked not to discharge patients until at the earliest 2 months post-treatment and to avoid using trauma-focused therapy for 6 months post-treatment.

The therapists included one man and seventeen women and varied in age (range 27–65) and treatment experience. All had completed their master's degrees, but about half were still in training to specialize in psychology or psychiatry. All had basic training in PE or EMDR or both; two had additional training in PE, and five had additional training/level two in EMDR.

The ward staff consisted of nurses (some specialized in psychiatric nursing) and social workers. All permanent ward staff had participated in an *in vivo* PE course and were responsible for the *in vivo* exposure part of the program. Therapists and representatives from ward staff had a daily 1-h meeting to ensure adherence to the treatment protocols (Foa et al., 2019; Shapiro, 2018), monitor progress, and plan the following sessions. On-site supervision in PE and EMDR therapy was provided by qualified supervisors to ensure treatment adherence.

All permanent staff and therapists who had participated in the first and/or second treatment program were invited to complete an anonymous questionnaire about how participation in the project had affected them as trauma therapists/ward staff. Of the eighteen contributing therapists, fifteen had participated in the treatment at the time of the survey. All therapists, except those involved in planning or supervising the study (5), were invited to complete a questionnaire about readiness to start TFT. Thus, all available therapists responded to the survey.

### 2.4 Psychometric measures

#### 2.4.1 PTSD Checklist for DSM-5 (PCL-5)

PTSD symptom severity was assessed through self-report using the PTSD Checklist for DSM-5 (PCL-5) (Blevins et al., 2015) at pre- and post-treatment, and at the 2- and 6-month follow-ups. The PCL-5 is a 20-item questionnaire that measures a broad range of PTSD symptoms experienced over the preceding month (alternatively, week), including re-experiencing, avoidance, and hypervigilance. Each item is rated on a five-point scale (0–4), with higher scores indicating greater symptom severity. The total score is calculated by summing responses across all items (range: 0–80). The reliable change index (RCI) was set at 10, as suggested by the National Center for PTSD (2013).

#### 2.4.2 The International Trauma Questionnaire (ITQ)

The International Trauma Questionnaire (ITQ) was used to determine whether patients met the ICD-11 diagnostic criteria for CPTSD. It was completed at baseline and at the 6-month follow-up. The scale is considered a valid instrument for assessing these criteria (Cloitre et al., 2018). The scale consists of six symptom clusters, all of which must be present and cause impairment in at least one area of daily life (i.e., work, school, or social life) to qualify for a CPTSD diagnosis. The symptom clusters include three PTSD symptom categories (re-experiencing, avoidance, and sense of threat) and three Disturbance of Self-Organization (DSO) symptom clusters (affective dysregulation, disturbances in relationships, and negative self-concept). A symptom cluster (consisting of two symptoms) is considered present when the score is ≥ 2 for both symptoms on a scale ranging from zero (not at all) to four (extremely). For both PTSD and CPTSD diagnosis, one of two symptoms from each symptom cluster and additional functional impairment are required. The total severity of PTSD and DSO symptom scores is calculated by summing items 1–6 (PTSD) and 7–12 (DSO), with a total score ranging from 0–48 (PTSD + DSO). In addition, the three DSO symptom clusters and the three PTSD symptom clusters separately have an overall score ranging from 0–8, with a total DSO or PTSD symptom score ranging from 0–24.

#### 2.4.3 The stressful life events screening questionnaire (SLESQ)

The Stressful Life Events Screening Questionnaire (SLESQ) (Goodman et al., 1998) was used to assess lifetime exposure to traumatic events prior to treatment. It is a 13-item self-report questionnaire that contains eleven specific and two general categories of events, including life-threatening accidents, physical and sexual abuse, and witnessing another person being killed or assaulted. Respondents are asked to indicate their experiences (“yes” or “no”). The questionnaire was used prior to treatment.

#### 2.4.4 The posttraumatic cognitions inventory (PTCI)

The Posttraumatic Cognitions Inventory (PTCI) (Foa et al., 1999) is a 36-item self-report questionnaire that assesses trauma-related negative cognitions and yields scores on three subscales (each item rated on a 1–7 Likert scale): negative cognitions about self, negative cognitions about the world, and negative cognitions about self-blame. The PTCI was administered at baseline, post-treatment, and at two- and six-month follow-up. The subscales demonstrate good internal consistency, test–retest reliability, and convergent validity with other psychometric assessments of trauma-related negative cognitions (Foa et al., 1999).

#### 2.4.5 Clinical outcomes in routine evaluation–outcome measure (CORE-OM)

Symptoms of psychological distress were assessed using the Clinical Outcomes in Routine Evaluation–Outcome Measure (CORE-OM) at baseline, post-treatment, and at two- and six-month follow-up. It measures symptoms across four domains: subjective wellbeing, problems and symptoms, functioning, and risk to self or others (Evans et al., 2002). The scale includes 34 items, each rated from zero (not at all) to four (severely).

#### 2.4.6 Work and social adjustment scale (WSAS)

Functioning was assessed using the Work and Social Adjustment Scale (WSAS) (Mundt et al., 2002) at baseline and at two- and six-month follow-up. WSAS consists of five items that measure impairment in work, social leisure activities, private leisure activities, home management, and close relationships. Patients rate the extent to which their mental health difficulties have interfered with each domain on a scale from 0 (not at all) to 8 (severely). The total score is 40. WSAS has shown high internal reliability and sensitivity to treatment effects (Zahra et al., 2014).

#### 2.4.7 Satisfaction with treatment

To measure satisfaction with treatment and individual treatment components, our research group created a simple eight-item questionnaire in which participants rated the individual treatment components (PE-imaginal exposure, PE-in-vivo, EMDR, Listening to PE recording, Psychoeducational group, Physical exercise, therapist rotation, and an evaluation of the treatment program in its totality) on a scale from one (very dissatisfied) to five (very satisfied). The questionnaire was used at the post-treatment assessment.

#### 2.4.8 Therapists and ward staff–Readiness to start TFT

A nine-item questionnaire (a therapist version and a ward staff version) was developed to assess whether the project had influenced participating therapists and ward staff in their work with patients with PTSD. The questionnaire was based on a similar questionnaire created by Van Minnen et al. (2018).

### 2.5 Statistical analysis

All statistical analyses were conducted in SPSS version 29.0 (IBM, Armonk, NY, United States). Symptom and functioning questionnaires were administered at multiple time points. Because missing data on the main outcome measures were minimal (1.07%), we used repeated-measures ANOVA to examine changes in symptoms and functioning over time. Missing data were handled using backfill for missing questionnaires or larger parts of questionnaires, while single missing items were handled using the mean score on the given scale/questionnaire. Sphericity was tested with Mauchly's test. Where the assumption was violated, Greenhouse–Geisser corrections were applied. Symptom measures (PCL-5, PTCI, and CORE) were assessed at four time points, whereas the functioning measure (WSAS) was assessed at three. The ITQ was administered only at baseline and at the 6-month follow-up. Therefore, the total score and subscores were analyzed with paired-samples *t*-tests. Effect sizes were reported using Cohen's *d* for *t*-tests and partial eta squared for ANOVA.

## 3 Results

### 3.1 Sample description

The sample consisted of 18 women aged 19–45 years (mean age = 29). All had a history of multiple traumas, including childhood sexual and physical abuse, sexual and physical abuse in adulthood, domestic abuse, witnessing serious violence, terrorist attacks, systematic bullying, parental substance abuse and/or mental illness, and severe parental neglect. At baseline, fourteen patients met the criteria for CPTSD, and four met the criteria for PTSD. Fourteen patients (78%) had experienced sexual trauma. Ten (56%) had current comorbid psychiatric diagnosis, including depressive episodes (33%), eating disorders (6%), social anxiety disorder, personality disorder (avoidant and mixed) (17%), autism spectrum disorder (6%), and Attention-deficit/hyperactivity disorder (ADHD) (6%). Half the patients were using psychotropic medication, all of which were antidepressants. Detailed patient characteristics are presented in Table 1. Baseline mean outcome scores show that the sample had high levels of PTSD symptoms and severe impairment in functioning (see Table 2).

**Table 1:** Baseline characteristics of the patient sample.

Baseline characteristic	*N*	%
Female gender	18	100
European ethnicity	15	83
Working	1	6
Student	4	22
Social security benefits	13	72
Married/partner	6	33
Unmarried/no partner	12	66
Comorbid disorders	10	56
Depressive episode	6	33
Eating disorders	2	12
Personality disorder (avoidant/mixed)	3	17
Social anxiety disorder	1	6
Autism spectrum disorder	1	6
Attention-deficit/hyperactivity disorder	1	6
Antidepressant use	9	50
Post-traumatic stress disorder (PTSD)	4	22
Complex PTSD	14	78

**Table 2:** Changes in symptoms and functioning from baseline to 6-month follow-up, with mean scores and standard deviations.

Psychometric measures	Baseline	Post	2-months	6-months	*F*	*p*	*η^2^p*
PCL-5	57.5 (8.7)	42.9 (14.7)	36.2 (14.2)	35.7 (14.3)	18.6	< 0.001	0.52
PTCI	164.6 (33.9)	129.7 (38.8)	125.4 (43.9)	126.1 (43.0)	7.8	0.001	0.32
PTCI-SB	19.7 (7.3)	14.2 (6.6)	13.6 (6.8)	13.2 (7.0)	5.4	0.003	0.24
PTCI-NCS	91.9 (22.6)	72.7 (24.4)	71.2 (28.7)	71.1 (28,0)	6.5	0.002	0.28
PTCI-NCW	38.2 (6.5)	30.9 (8.9)	30.3 (9.9)	30.9 (8.7)	5.7	0.006	0.25
CORE	72.9 (14.2)	60.7 (15.7)	62.0 (19.2)	60.7 (14.9)	5.1	0.004	0.22
CORE-SWB	11.0 (2.6)	9.4 (3.0)	9.4 (2.6)	9.6 (2.0)	3.2	0.030	0.16
CORE- F	20.6 (5.3)	18.4 (4.2)	18.4 (7.0)	18.2 (4.6)	1.1	0.353	0.06
CORE-PS	34.2 (7.0)	26.7 (8.3)	26.9 (9.0)	26.1 (8.7)	9.5	< 0.001	0.36
CORE-R	3.1 (3.4)	2.6 (2.7)	3.4 (4.0)	2.7 (2.8)	0.6	0.636	0.03
WSAS	26.6 (6.8)	-	21.5 (6.9)	21.3 (9.0)	4.1	0.025	0.20

PCL-5, PTSD checklist for DSM-5; PTCI, posttraumatic cognitions inventory; PTCI-SB, self-blame subscale; PTCI-NCS, negative cognitions about the self subscale; PTCI-NCW, negative cognitions about the world subscale; CORE, clinical outcomes in routine evaluation; SWB, subjective wellbeing; PS, problems and symptoms; *F*, functioning; *R*, risk to self or others; WSAS, work and social adjustment scale.

### 3.2 Symptom and functioning changes

PTSD symptoms (PCL-5) declined significantly over time, *F*(3, 51) = 18.6, *p* < 0.001, η^2^*p* = 0.52. Mean scores dropped from 57.5 (SD = 8.7) at baseline to 35.7 (SD = 14.3) at 6-month follow-up. Clinically meaningful change was observed in 16 participants (89%) at 2-month follow-up and in 15 participants (83%) at 6-month follow-up. No participant experienced an overall worsening on the PCL-5. Trauma-related negative cognitions (PTCI) also decreased significantly, *F*(3, 51) = 7.8, *p* = 0.001, η^2^*p* = 0.32. Significant reductions were observed across all PTCI subscales. Psychological distress (CORE total) declined significantly, *F*(3, 51) = 5.1, *p* = 0.004, η^2^*p* = 0.22, with mean scores dropping from 72.9 (SD = 14.2) at baseline to 60.7 (SD = 14.9) at 6 months. Among the subscales, problems/symptoms showed the strongest reduction, *F*(3, 51) = 9.5, *p* < 0.001, η^2^*p* = 0.36. Subjective wellbeing also improved, *F*(3, 51) = 3.2, *p* = 0.030, η^2^*p* = 0.16, while reductions in functioning and risk were not statistically significant. Functioning measured by WSAS, however, showed a significant change across baseline, 2-month, and 6-month assessments, *F*(2, 34) = 4.1, *p* = 0.025, η^2^*p* = 0.20.

The International Trauma Questionnaire (ITQ) demonstrated large reductions in both PTSD and CPTSD symptoms from baseline to 6-month follow-up. Total ITQ scores decreased from 51.1 (SD = 9.7) at baseline to 33.2 (SD = 15.8) at 6 months, corresponding to a large effect size (*d* = 1.18). The PTSD subscale declined from 17.2 (SD = 4.2) to 9.3 (SD = 5.1), reflecting a very large effect (*d* = 1.56), and the DSO subscale decreased from 16.5 (SD = 4.5) to 11.6 (SD = 6.2), with a large effect (*d* = 0.94). At the diagnostic level, seven of the fourteen patients who met the CPTSD criteria at baseline were below the CPTSD cut-off at 6-month follow-up. All four participants with PTSD at baseline were below the diagnostic cut-off at 6 months.

### 3.3 Acceptability and satisfaction

All participants (*N* = 18) completed the program, with no dropouts during treatment or follow-up. Attendance was very high, with only one patient missing four sessions (due to an acute illness in a close family member). No participant experienced an overall worsening on the PCL-5. No significant adverse effects, including suicide attempts, or having to move a patient to a higher level of care, were reported or observed by clinical staff during treatment or in the follow-up period. These findings indicate that the program was acceptable to participants.

Table 3 presents patient satisfaction with the treatment program as a whole and with its individual components. Overall satisfaction with the treatment program was high (*M* = 4.47 on a 1–5 scale). Similarly, satisfaction was high across all treatment components (*M* = 3.76–4.59). EMDR and PE-Imaginal exposure received particularly high ratings, with narrow score ranges (3–5). Therapist rotation was also rated highly, though with greater variability (2–5). PE-listening to recorded sessions received the lowest ratings (*M* = 3.76) and showed the greatest variability (range 1–5).

**Table 3:** Satisfaction with the treatment program and the treatment components (*n* = 17).

Treatment satisfaction	Mean (1–5)	Range	Std. deviation
Overall satisfaction	4.47	(3–5)	0.72
PE–Imaginal exposure	4.53	(2–5)	0.80
PE–*In vivo* exposure	4.23	(2–5)	0.90
PE–Listening to recorded sessions	3.76	(1–5)	1.30
EMDR	4.59	(3–5)	0.62
Psychoeducational group	4.18	(2–5)	0.95
Physical exercise	4.00	(2–5)	0.94
Therapist rotation	4.06	(2–5)	0.75

PE, prolonged exposure, EMDR, eye movement desensitization and reprocessing.

### 3.4 Therapists and ward staff–Readiness to provide TFT

Table 4 presents the therapists' and ward staff's responses to a questionnaire about whether participating in the intensive trauma treatment program had influenced their readiness to provide TFT. All therapists and 90% of ward staff reported that they were more likely to provide TFT to patients, even those with significant comorbidity, and that they would assess patients as ready for TFT earlier than before, rather than proceeding with stabilization. Most therapists and ward staff reported being less fearful of making patients worse by providing TFT, and that they now dared to address avoidance behavior and stick to exposure work. 70% of therapists and 90% of ward staff reported providing more TFT outside the project than before participating in the project.

**Table 4:** Readiness to start TFT: therapists and ward staff.

Questions to therapists (*N* = 10)/questions to ward staff (*N* = 10)	Therapists			Ward staff		
	Agree	No change	Disagree	Agree	No change	Disagree
In the future, I am more likely to treat PTSD patients with Prolonged Exposure (PE) or EMDR, even when there is significant comorbidity/In the future, I will be more likely to treat PTSD patients using *in vivo* exposure, even in cases with significant comorbidity	100% (10)			90% (9)	10% (1)	
In my future therapeutic work, I will be more likely to assess patients as ready for trauma processing (rather than stabilization)/I anticipate being more confident in assessing patients as ready for *in vivo* exposure	100% (10)			90% (9)	10% (1)	
Currently, within the project, I am conducting PE and EMDR with patients I previously would not have dared to work with in this way/Currently, within the project, I am conducting *in vivo* exposure with patients I previously would not have dared to approach in this way	80% (8)	20% (2)		80% (8)	20% (2)	
My fear of making patients worse has decreased	90% (9)		10% (1)	80% (8)	20% (2)	
I feel that I have been given too much responsibility in the project		10% (1)	90% (9)	10% (1)	20% (2)	70% (7)
I now adhere more closely to the treatment manual/I now adhere more closely to the *in vivo* exposure manual	40% (4)	40% (4)	20% (2)	90% (9)	10% (1)	
I do not deviate from the manual	10% (1)	40% (4)	50% (5)	50% (5)	30% (3)	20% (2)
I now dare to address avoidance behavior to a greater extent and stick with the exposure work	80% (8)	10% (1)	10% (1)	80% (8)	10% (1)	10% (1)
Outside the ITBD project, I am now providing more trauma-focused treatment (rather than stabilization)/Outside the project, I am now increasingly providing exposure-focused treatment	70% (7)	30% (3)		90% (9)		10% (1)

## 4 Discussion

This study examined the feasibility of an eight-day intensive, multicomponent trauma treatment program delivered in a facility not specialized in trauma treatment and staffed by several therapists with limited TFT experience, to determine whether the program would produce clinically meaningful change in PTSD symptoms. Our results show that no patient experienced a worsening of PTSD symptoms or significant adverse effects, and no patients dropped out of the treatment program. Additionally, we found a significant reduction in PTSD symptoms, with 83% of our sample experiencing clinically meaningful change from baseline to 6-month follow-up. Furthermore, half of our patients with CPTSD and all our patients with PTSD (most with comorbid disorders) lost their diagnosis according to the ITQ standard.

Our results are similar to those of Auren et al. (2022) who found that 62% of patients had lost their PTSD diagnosis at 3-month follow-up according to PCL-5 standard. However, this study did not report whether patients had CPTSD, and its sample had a lower baseline PCL-5 score (*M* = 49.79). Our scores are poorer than those reported by Gahnfelt et al., whose sample had baseline PCL-5 scores similar to ours. They reported a 100% loss of diagnosis at 3-month follow-up according to ICD-11 criteria, and 100% experienced clinically meaningful change according to PCL-5 measures. However, this sample had a lower proportion of CPTSD (27%), and one of twelve participants dropped out of the treatment (Gahnfelt et al., 2023). Voorendonk et al., who reported results from a much larger sample (*N* = 308) at a very specialized trauma treatment center, reported that 87.7% of patients (*N* = 203) with an ITQ-based CPTSD diagnosis lost their diagnosis post-treatment. 85.4% of the total sample was reported to have experienced clinically meaningful change, as measured by PCL-5 scores (Voorendonk et al., 2020).

The current study also found a statistically significant reduction in trauma-related negative self-cognitions, including self-blame. This aligns with a study by Holiday et al. of veterans with military sexual trauma-related PTSD who received weekly CPT sessions. The study found that only changes in self-blame predicted and temporally preceded changes in PTSD symptoms from baseline to 6-month follow-up, highlighting a potential mechanism of change (Holliday et al., 2018). It also resonates with our two previous qualitative studies, in which patients described greater acceptance and ownership of their trauma history and a clearer sense of not being responsible for the traumatic events they had previously blamed themselves for (Vaage-Kowalzik et al., 2024, 2025b).

Our sample reported high treatment satisfaction with the overall treatment program. Auren et al. similarly found high levels of patient satisfaction on the Client Satisfaction Questionnaire (CSQ-8) (Auren et al., 2022). In a survey by Van Minnen et al., only 14% of patients answered ‘yes' to the question ‘Would you have preferred being treated by one therapist instead of a rotating team of therapists?', indicating high levels of patient satisfaction with TR (Van Minnen et al., 2018). We also found high levels of satisfaction with TR, which align with our qualitative study results (Vaage-Kowalzik et al., 2024, 2025b).

Our sample also reported high satisfaction with the trauma-processing elements of the treatment program, with similarly high scores for EMDR and imaginal exposure (PE). In an article exploring the combination and sequencing of EMDR and PE, Van Minnen et al. found that patients who received PE first and EMDR second showed a significantly greater reduction in PTSD symptoms compared to patients who received EMDR first and PE second, and that patients rated EMDR and PE sessions as equally helpful (Van Minnen et al., 2020).

The treatment component with the lowest score was *listening to PE recordings;* this was also the treatment element that received the largest score range (from 1–5). In qualitative interviews, we have reported that some patients gained new insights and perspectives from listening to the session recording, whereas others found this aspect of the program a strain and not helpful (Vaage-Kowalzik et al., 2024, 2025b).

No patients dropped out of the current study, and participation in the individual treatment elements was high. Auren et al. reported a 3% loss of attendance in PE and EMDR sessions (Auren et al., 2022), which is more than twice that of the current study. A higher number of attended sessions could be a benefit of delivering ITFT in an inpatient facility.

Regarding the project's impact on participating therapists and ward staff, we found that it appeared to make therapists less fearful of making patients deteriorate from TFT and more ready to address avoidance patterns. Participating in the project seems to increase therapists' and ward staff's readiness to commence TFT and to consider even patients with considerable comorbidities as candidates for TFT rather than stabilization. Our findings are in line with a similar survey conducted by Van Minnen et al., which suggests that gaining more clinical experience in the safe context of a therapist rotation may facilitate easier implementation of TFT (Van Minnen et al., 2018).

### 4.1 Strengths and limitations

This study has several important limitations. It includes only female participants, primarily patients of European ethnicity, which limits its generalizability. The results also cannot be generalized to outpatient settings or to clinics without supervision resources or staff with TFT qualifications. Furthermore, as a feasibility study, it includes a smaller number of participants, which limits the conclusions that can be drawn from statistical analysis. Notably, the study lacks a control group, making it difficult to draw firm conclusions about the treatment's effects. We were also unable to control for the possible effects of therapy received after the treatment ended; however, the outpatient therapists were asked to avoid providing TFT during the follow-up period. Adverse events were carefully monitored by staff during treatment but were not systematically assessed throughout the follow-up period. Lastly, treatment satisfaction was measured using a simple questionnaire designed by our research group to fit the multimodal treatment program; however, this questionnaire has not been validated, and its results must thus be interpreted with caution.

## 5 Conclusions and recommendations

In conclusion, our findings suggest that intensive trauma-focused treatment is feasible in a non-trauma-specialized public treatment facility staffed by several therapists with limited experience with TFT, if the appropriate basic training and supervision resources are available. The reduction in PTSD symptoms was considerable and sustained at the 6-month follow-up, and the majority of patients no longer scored above the diagnostic criteria for PTSD/CPTSD. Trauma-related negative cognitions also showed a statistically significant reduction. Treatment satisfaction was high, including measures of the individual treatment components, such as TR. Importantly, all participants completed treatment, and no significant adverse effects were reported. Furthermore, our therapist survey indicates that participating in an intensive trauma treatment program with TR seems to reduce therapists' and ward staff's avoidance and increase their readiness to provide TFT.

Clinical implications: our findings suggest that facilities not specialized in trauma treatment can implement intensive inpatient trauma treatment with TR. Treatment programs that include TR may increase therapists' and ward staff's readiness to provide TFT to patients with significant comorbidity and complex trauma histories. Because not all patients in our sample experienced clinically significant improvement, a better understanding of who is suitable for intensive trauma treatment and who requires other treatment options is necessary.

Future research recommendations: future research should examine the long-term efficacy of intensive trauma treatment programs beyond a 6-month follow-up. More attention should be given to both the treatment benefits and the experiences of male patients and patients from minority backgrounds. The individual and combined effects of the different treatment elements should be further explored to optimize the treatment program.

## Data Availability

The raw data supporting the conclusions of this article will be made available by the authors, without undue reservation.

## References

[B1] Auren T. J. B. Klæth J. R. Jensen A. G. Solem S. (2022). Intensive outpatient treatment for PTSD: an open trial combining prolonged exposure therapy, EMDR, and physical activity. Eur. J. Psychotraumatol. 13:2128048. doi: 10.1080/20008066.2022.2128048 36237826 PMC9553174

[B2] Blevins C. A. Weathers F. W. Davis M. T. Witte T. K. Domino J. L. (2015). The Posttraumatic Stress Disorder Checklist for DSM-5 (PCL-5): development and initial psychometric evaluation. J. Trauma Stress 28, 489–498. doi: 10.1002/jts.22059 26606250

[B3] Cloitre M. Shevlin M. Brewin C. R. Bisson J. I. Roberts N. P. Maercker A. . (2018). The International Trauma Questionnaire: development of a self-report measure of ICD-11 PTSD and complex PTSD. Acta Psychiatr. Scand 138, 536–546. doi: 10.1111/acps.12956 30178492

[B4] Craig C. D. Sprang G. (2010). Compassion satisfaction, compassion fatigue, and burnout in a national sample of trauma treatment therapists. Anxiety Stress Coping 23, 319–339. doi: 10.1080/10615800903085818 19590994

[B5] Eastwood O. Peters W. Cohen J. Murray L. Rice S. Alvarez-Jimenez M. . (2021). “Like a huge weight lifted off my shoulders”: exploring young peoples' experiences of treatment in a pilot trial of trauma-focused cognitive behavioral therapy. Psychother Res. 31, 737–751. doi: 10.1080/10503307.2020.1851794 33283674

[B6] Evans C. Connell J. Barkham M. Margison F. McGrath G. Mellor-Clark J. . (2002). Towards a standardised brief outcome measure: psychometric properties and utility of the CORE-OM. Br. J. Psychiatry 180, 51–60. doi: 10.1192/bjp.180.1.51 11772852

[B7] Finch J. Ford C. Grainger L. Meiser-Stedman R. (2020). A systematic review of the clinician related barriers and facilitators to the use of evidence-informed interventions for post traumatic stress. J. Affect Disord. 263, 175–186. doi: 10.1016/j.jad.2019.11.143 31818775

[B8] First M. B. (1997). User's Guide for the Structured Clinical Interview for DSM-IV Axis II Personality Disorders: SCID-II. Washington, DC: American Psychiatric Publishing.

[B9] Foa E. B. Ehlers A. Clark D. M. Tolin D. F. Orsillo S. M. (1999). The Posttraumatic Cognitions Inventory (PTCI): development and validation. Psychol. Assess. 11, 303–314. doi: 10.1037/1040-3590.11.3.303

[B10] Foa E. B. Hembree E. A. Rothbaum B. O. (2019). “*Prolonged exposure therapy for PTSD: emotional processing of traumatic experiences*,” in Therapist Guide, 2nd Edn, eds. E. B. Foa, E. A. Hembree, B. O. Rothbaum, S. A. M. Rauch. Oxford: Oxford University Press. doi: 10.1093/med-psych/9780190926939.001.0001

[B11] Gahnfelt H. Carlsson P. F. G. Blomdahl C. (2023). Is it safe enough? A pilot feasibility study of an 8-day intensive treatment for severe PTSD [Brief Research Report]. Front. Psychiatr. 1:4. doi: 10.3389/fpsyt.2023.1200411 37547221 PMC10397389

[B12] Goodman L. A. Corcoran C. Turner K. Yuan N. Green B. L. (1998). Assessing traumatic event exposure: general issues and preliminary findings for the Stressful Life Events Screening Questionnaire. J. Traumatic Stress 11, 521–542. doi: 10.1023/A:1024456713321 9690191

[B13] Holliday R. Holder N. Surís A. (2018). Reductions in self-blame cognitions predict PTSD improvements with cognitive processing therapy for military sexual trauma-related PTSD. Psychiatr. Res. 263, 181–184. doi: 10.1016/j.psychres.2018.03.007 29573657

[B14] Hoogeveen M. De Jongh A. Matthijssen A. S. Voorendonk E. M. (2026). Therapeutic alliance in intensive PTSD treatment with a therapist rotation model. Eur. J. Psychotraumatol. 17:2664272. doi: 10.1080/20008066.2026.2664272 42246501 PMC13244515

[B15] Kolthof K. Voorendonk E. Van minnen A. De Jongh A. (2022). Effects of intensive trauma-focused treatment of individuals with both post-traumatic stress disorder and borderline personality disorder. Eur. J. Psychotraumatol. 13:2143076. doi: 10.1080/20008066.2022.2143076 38872595 PMC9704092

[B16] Lewis C. Roberts N. P. Gibson S. Bisson J. I. (2020). Dropout from psychological therapies for post-traumatic stress disorder (PTSD) in adults: systematic review and meta-analysis. Eur. J. Psychotraumatol. 11:1709709. doi: 10.1080/20008198.2019.1709709 32284816 PMC7144189

[B17] Mundt J. C. Marks I. M. Shear M. K. Greist J. H. (2002). The Work and Social Adjustment Scale: a simple measure of impairment in functioning. Br. J. Psychiatry 180, 461–464. doi: 10.1192/bjp.180.5.461 11983645

[B18] National Center for PTSD (2013). Assessment: PTSD checklist for DSM-5 (PCL-5). Available online at: https://www.ptsd.va.gov/professional/assessment/adult-sr/ptsd-checklist.asp (Accessed April 14, 2013).

[B19] Paridaen P. Voorendonk E. M. Gomon G. Hoogendoorn E. A. van Minnen A. De Jongh A. . (2023). Changes in comorbid depression following intensive trauma-focused treatment for PTSD and complex PTSD. Eur. J. Psychotraumatol. 14:2258313. doi: 10.1080/20008066.2023.2258313 37796651 PMC10557564

[B20] Ragsdale K. A. Watkins L. E. Sherrill A. M. Zwiebach L. Rothbaum B. O. (2020). Advances in ptsd treatment delivery: evidence base and future directions for intensive outpatient programs. Curr. Treatment Options Psychiatr. 7, 291–300. doi: 10.1007/s40501-020-00219-7

[B21] Sciarrino N. A. Warnecke A. J. Teng E. J. (2020). A systematic review of intensive empirically supported treatments for posttraumatic stress disorder. J. Trauma Stress 33, 443–454. doi: 10.1002/jts.22556 32598561

[B22] Shapiro F. (2018). Eye Movement Desensitization And Reprocessing (EDMR) Therapy: Basic Principles, Protocols, And Procedures, 3rd Edn. New York, NY: The Guilford Press.

[B23] Sheehan D. V. Lecrubier Y. Sheehan K. H. Amorim P. Janavs J. Weiller E. . (1998). The Mini-International Neuropsychiatric Interview (M.I.N.I.): the development and validation of a structured diagnostic psychiatric interview for DSM-IV and ICD-10. J. Clin. Psychiatr. 59, 22–33. doi: 10.1016/S0924-9338(97)83297-X 9881538

[B24] Sodeke-Gregson E. A. Holttum S. Billings J. (2013). Compassion satisfaction, burnout, and secondary traumatic stress in UK therapists who work with adult trauma clients. Eur. J. Psychotraumatol. 4:3402. doi: 10.3402/ejpt.v4i0.21869 24386550 PMC3877781

[B25] Vaage-Kowalzik V. Engeset J. Jakobsen M. Andreassen W. Evensen J. H. (2024). Exhausting, but necessary: the lived experience of participants in an intensive inpatient trauma treatment program [Clinical Trial]. Front. Psychol. 15:1341716. doi: 10.3389/fpsyg.2024.1341716 38863672 PMC11165995

[B26] Vaage-Kowalzik V. Engeset J. Jakobsen M. Evensen J. H. (2025a). Educational, but demanding: the experience of therapists in an intensive inpatient trauma treatment program [Original Research]. Front. Psychol. 16:2025. doi: 10.3389/fpsyg.2025.1581055 40949339 PMC12426260

[B27] Vaage-Kowalzik V. Engeset J. Jakobsen M. Evensen J. H. (2025b). The world does not look the same anymore: the experiences of patients six months after finishing an intensive trauma treatment program. BMC Psychol. 14:77. doi: 10.1186/s40359-025-03826-2 41382171 PMC12809926

[B28] Van Minnen A. Hendriks L. Kleine R. Hendriks G. J. Verhagen M. De Jongh A. . (2018). Therapist rotation: a novel approach for implementation of trauma-focused treatment in post-traumatic stress disorder. Eur. J. Psychotraumatol. 9:1492836. doi: 10.1080/20008198.2018.1492836 30034642 PMC6052418

[B29] Van Minnen A. Voorendonk E. M. Rozendaal L. De Jongh A. (2020). Sequence matters: combining prolonged exposure and EMDR therapy for PTSD. Psychiatr. Res. 290:113032. doi: 10.1016/j.psychres.2020.113032 32454314

[B30] Van Woudenberg C. Voorendonk E. M. Bongaerts H. Zoet H. A. Verhagen M. Lee C. W. . (2018). Effectiveness of an intensive treatment programme combining prolonged exposure and eye movement desensitization and reprocessing for severe post-traumatic stress disorder. Eur. J. Psychotraumatol. 9:1487225. doi: 10.1080/20008198.2018.1487225 30013726 PMC6041781

[B31] Voorendonk E. M. De Jongh A. Rozendaal L. Van Minnen A. (2020). Trauma-focused treatment outcome for complex PTSD patients: results of an intensive treatment programme. Eur. J. Psychotraumatol. 11:1783955. doi: 10.1080/20008198.2020.1783955 33029323 PMC7473266

[B32] Wagenmans A. Agnes V. M. Marieke S. De Jongh A. (2018). The impact of childhood sexual abuse on the outcome of intensive trauma-focused treatment for PTSD. Eur. J. Psychotraumatol. 9:1430962. doi: 10.1080/20008198.2018.1430962 29441153 PMC5804725

[B33] Weber M. Schumacher S. Hannig W. Barth J. Lotzin A. Schäfer I. . (2021). Long-term outcomes of psychological treatment for posttraumatic stress disorder: a systematic review and meta-analysis. Psychol. Med. 51, 1420–1430. doi: 10.1017/S003329172100163X 34176532 PMC8311818

[B34] Zahra D. Qureshi A. Henley W. Taylor R. Quinn C. Pooler J. . (2014). The work and social adjustment scale: reliability, sensitivity and value. Int. J. Psychiatr. Clin. Pract. 18, 131–138. doi: 10.3109/13651501.2014.894072 24527886

[B35] Zoet H. A. Wagenmans A. van Minnen A. De Jongh A. (2018). Presence of the dissociative subtype of PTSD does not moderate the outcome of intensive trauma-focused treatment for PTSD. Eur. J. Psychotraumatol. 9:1468707. doi: 10.1080/20008198.2018.1468707 29805779 PMC5965028

