# Supplementary material for: Intensive trauma-focused treatment for PTSD and Complex PTSD in a public clinic: a feasibility study
Source: Front Psychol. 2026 Sep 9;17:1869412. doi: 10.3389/fpsyg.2026.1869412 (PMC13597857; doi:10.3389/fpsyg.2026.1869412)
Supplement: Supplementary file 1 [file Data_Sheet_1.pdf]

Appendix 1. Details of the treatment program.

The two-week treatment program included eight full treatment days (Monday through Thursday) and two days (Friday) for team meetings and planning. The treatment facility is a five-day ward open Monday through Friday, and patients went home during the mid-treatment weekend. The program included daily PE (imaginal exposure and in vivo exposure) and EMDR sessions, with physical activity (PA) between sessions and psychoeducational groups (see fig. 1). The combination of EMDR and PE is based on the assumption that interventions with different mechanisms of action could be more effective than a single treatment method (Bryant et al., 2008). The program is modeled on the PSYTREC trauma treatment program in the Netherlands (Van Woudenberg et al., 2018) but includes less physical activity and shorter EMDR sessions. Rather than focusing on physical activity, we have, similarly to the Trondheim trauma treatment program (Brynhildsvoll Auren et al., 2021), expanded elements of PE by incorporating listening to session recordings of imaginal exposure (IE) and in vivo exposure (IVE). IVE was emphasized in the treatment program because this element targets avoidance behavior, which is particularly relevant for patients with PTSD (Cusack et al., 2016).

A multidisciplinary team was assigned to each patient. Therapist rotation was used for EMDR and Imaginal Exposure (part of PE). As a result, patients engaged with 11-12 different therapists during treatment. IE and EMDR were delivered by psychologists and psychiatrists trained in these methods. The treatment ward had three therapists with this training, and therapists with relevant training thus had to be “borrowed” from other wards and from the outpatient clinic at the treatment facility. IVE, PA, PEG, and, when needed, assistance while listening to recorded PE sessions were provided by ward staff. Ward staff were also available for supportive ad hoc counseling. Ward staff consisted of nurses (some specialized in psychiatric nursing) and social workers. All permanent staff had completed trauma treatment

courses, including a course in PE. Therapists and representatives from ward staff held a daily one-hour meeting to monitor progress, plan the following sessions, and ensure adherence to the treatment protocols.

Figure 1. Daily treatment program

**Daily treatment program**
**09.00 – 10.00** Imaginal exposure (PE)
**10.30 – 12.00** In vivo exposure therapy (PE)
**12.45 – 13.40** Physical activity/sports
**14.00 – 15.00** EMDR
**15.30 – 15.45** Afternoon meeting
**16.15 – 17.00** Psychoeducational group
**18.00 – 19.00** Listening to recorded PE session
Evenings: patients encouraged to socialize in common area: do puzzles, boardgames etc.
The program includes breaks for meals and snacks.

References:

Bryant, R. A., Moulds, M. L., Guthrie, R. M., Dang, S. T., Mastrodomenico, J., Nixon, R. D., Felmingham, K. L., Hopwood, S., & Creamer, M. (2008). A randomized controlled trial of exposure therapy and cognitive restructuring for posttraumatic stress disorder. *J Consult Clin Psychol*, *76*(4), 695-703. https://doi.org/10.1037/a0012616

Brynhildsvoll Auren, T. J., Gjerde Jensen, A., Rendum Klæth, J., Maksic, E., & Solem, S. (2021). Intensive outpatient treatment for PTSD: a pilot feasibility study combining prolonged exposure therapy, EMDR, physical activity, and psychoeducation. *Eur J Psychotraumatol*, *12*(1), 1917878. https://doi.org/10.1080/20008198.2021.1917878

Cusack, K., Jonas, D. E., Forneris, C. A., Wines, C., Sonis, J., Middleton, J. C., Feltner, C., Brownley, K. A., Olmsted, K. R., Greenblatt, A., Weil, A., & Gaynes, B. N. (2016). Psychological treatments for adults with posttraumatic stress disorder: A systematic review and meta-analysis. *Clin Psychol Rev*, *43*, 128-141. https://doi.org/10.1016/j.cpr.2015.10.003

Van Woudenberg, C., Voorendonk, E. M., Bongaerts, H., Zoet, H. A., Verhagen, M., Lee, C. W., van Minnen, A., & De Jongh, A. (2018). Effectiveness of an intensive treatment programme combining prolonged exposure and eye movement desensitization and reprocessing for

severe post-traumatic stress disorder. *Eur J Psychotraumatol*, *9*(1), 1487225.
https://doi.org/10.1080/20008198.2018.1487225
